# The Promise of Radiotherapy in High-Risk Non-Muscle Invasive Bladder Cancer

**DOI:** 10.3390/cancers17040628

**Published:** 2025-02-13

**Authors:** Becky Bola, Peter J. Hoskin, Vijay Sangar, Ananya Choudhury

**Affiliations:** 1 Mersey and West Lancashire Teaching Hospitals Trust, Whiston Hospital, Warrington Road, Prescot L35 5DR, UK; 2 The Genito Urinary Cancer Group, The Christie NHS Foundation Trust, 550 Wilmslow Road, Manchester M20 4BX, UK; 3 Translational Radiobiology Group, Division of Cancer Sciences, The Oglesby Cancer Research Building, The University of Manchester, 555 Wilmslow Road, Manchester M20 4GJ, UK; 4 The Department of Clinical Oncology, The Christie NHS Foundation Trust, 550 Wilmslow Road, Manchester M20 4BX, UK; 5 Manchester Foundation Trust, Wythenshawe Hospital, Southmoor Road, Wythenshawe, Manchester M23 9LT, UK

**Keywords:** NMIBC, radiotherapy, bladder cancer, non-muscle invasive bladder cancer

## Abstract

Global shortages, toxicities, and high levels of incomplete treatment with Bacillus Calmette Guerin (BCG) for non-muscle invasive bladder cancer has resulted in increasing interest in alternative treatments. Radiotherapy is not the standard of care for non-muscle invasive bladder cancer (NMIBC), despite being routinely used in muscle invasive bladder cancer. Modern techniques and advances in technology mean that radiotherapy can be delivered with increased precision in reducing normal tissue damage. Developing novel biomarker approaches, together with combination approaches with radiosensitisers and other systemic treatments, means that radiotherapy could offer greater benefits than current treatments with BCG or surgery. This review summarises the current landscape and future potential of radiotherapy for high-risk NMIBC.

## Simple Summary

Non-muscle invasive bladder cancer (NMIBC) is not currently treated with radiotherapy. However, other bladder cancers are successfully treated with radiotherapy. In this review, we discuss how NMIBC is treated currently and what the advantages and disadvantages of these treatments are. We summarise the recent developments in the treatment of NMIBC and highlight the promise of radiotherapy in this context. We then discuss future treatments for non-muscle invasive bladder cancer and how existing treatments can be made more effective.

## 1. Introduction

Bladder cancer is the 11th most common cancer in the UK, and there are around 10,500 new cases every year (2017–2019) [1]. Of those diagnosed, 56% are aged over 75, and incidence rates are highest in the 85–89-year age group. Bladder cancer is classified into muscle invasive (31%) and non-muscle invasive (69%) forms [2]. NMIBC comprises tumour stages CIS (carcinoma in situ), Ta, and T1 and then is further categorised into low, intermediate, high risk, or very high risk (Table 1) [3].

The standard of care for high-risk NMIBC is BCG, the efficacy of which has excellent (1a) evidence; it has been shown in five meta-analyses to be superior to both TURBT alone and TURBT + chemotherapy at preventing tumour recurrence. Specifically, BCG in intermediate to high-risk NMIBC reduces the risk of the recurrence of NIMBC to between 14 and 26% and the risk of progression to muscle invasive bladder cancer to approximately 20% [4], a finding supported by two metanalyses [5]. BCG has some limitations; it has a higher risk of side effects than intravesical chemotherapy, and there is a risk of delayed hypersensitivity which can occur many years after the completion of treatment. These adverse events contribute to a relatively high rate of dose reduction or the non-completion of the maintenance treatment (in one study, only 19% received all maintenance installations) [6]. There is also a 1% risk of BCG infection [5], which means that this treatment is not recommended in immunocompromised individuals [5]. Despite these limitations and the recent worldwide shortages [5], BCG represents an excellent treatment option for most NMIBC suffers; if this is unsuccessful, then these patients typically receive radical cystectomy.

Early radical cystectomy (patients receiving cystectomy before the development of muscle invasive disease [5,7,8,9]) leads to a disease-free survival (DFS) rate of 80%. TURBT is unreliable as a staging modality as 27 to 51% of patients thought to have NMIBC are found to have invasive disease at cystectomy [3,10,11,12], with a further 15% found to have lymph node metastases [13,14]. Early radical cystectomy is frequently accompanied by an increased risk of sexual dysfunction, malabsorption, and urinary infection, as well as the acute surgical risks of infection, bleeding, and anastomotic leakage [15]. For many, this is an unacceptably high morbidity burden. As standard treatments for NMIBC are limited to topical and surgical approaches, these patients are under-served in terms of treatment options.

## 2. Current Use of Radiotherapy

Most large-scale studies evaluating the use of radiotherapy in transitional cell carcinoma are conducted in muscle invasive bladder cancer (MIBC). MIBC international guidelines recommend radiotherapy to be considered as an alternative to surgery in patients with organ-confined muscle invasive bladder cancer [16,17]. In this context, trimodality therapy (TURBT, chemotherapy and radiotherapy) is considered to have equivalent outcomes to radical surgery. In contemporary series, trimodality therapy is equivalent to or better than radical cystectomy in all outcome measures considered (metastasis-free survival, cancer specific survival, and overall survival) [18,19]. Despite these data, radiotherapy does not currently form part of the accepted treatment regime in NMIBC. One reason for this is that most of the existing data for radiotherapy in NMIBC have small sample sizes and inconsistent methodology. A recent systemic review of 13 small-scale studies in high-grade T1 or T1 bladder cancer with high-risk features showed that ten studies used radiotherapy alone and three used brachytherapy in addition to radiotherapy. These analyses suggested that the rates of complete response, progression, and recurrence with radiotherapy were equivalent to those seen with the standard of care (TURBT followed by intravesical therapy with BCG), although there was a high degree of variation between studies. A total of 78.2% of patients in the pooled analysis demonstrated complete response with radiotherapy, while invasive disease progression was low at 17% (95% CI 12–21%) and NMIBC recurrence was 30% (95% CI 20.4–39.2%) [4]. A similar meta-analysis of treatment with BCG vs. mitomycin following TURBT suggested that there was a recurrence rate of 38.6% with BCG and 46.4% with mitomycin C [20].

The UK conducted the largest multicentre randomised control trial which evaluated whether radiotherapy reduced the progression of NMIBC to invasive disease. Harland et al. followed 210 patients in two cohorts over 11 years [21]. One cohort (77 patients) had T1G3 NxM0 with unifocal disease, and this cohort was either observed or received radiotherapy following surgical excision of the tumour. The other had CIS or multifocal disease and received either standard-of-care intravesical treatment or radiotherapy. The study demonstrated equivalence between both approaches, with hazard ratios for progression-free interval at 1.07 (95% CI 0.65–1.74), progression-free survival at 1.35 (95% CI 0.91–1.98), and overall survival at 1.32 (95% CI 0.86–2.04) [21]. The authors concluded that radiotherapy was no better than BCG for high-risk NMIBC and that the outcomes in this cohort were poor regardless of the treatment offered [21]. This study had some limitations. One cohort only had 77 patients, which was then further subdivided into a radiotherapy and an observation group. Also, as the study was conducted over 37 centres and patients were recruited over an 11-year period, it was likely challenging to ensure treatment parity over multiple locations and time periods.

In 2006, Weiss et al. demonstrated that radiotherapy or chemoradiotherapy following TURBT had similar efficacy to intravesical treatment or early cystectomy in a cohort of patients with high-risk T1 bladder cancer [22]. These patients received TURBT and either radiotherapy or platinum-based chemoradiotherapy and a restaging TURBT 6 weeks later. If disease progressed following these interventions, then salvage cystectomy was performed. In total, 121/137 achieved complete response, with >80% of patients preserving their bladder [22].

Patients who progress following TURBT and intravesicular biologic or chemotherapy are typically offered cystectomy. Dahl et al. evaluated the efficacy of a trimodality bladder-sparing approach in their single-arm phase II trial [23]. They recruited patients with BCG-unresponsive disease. Patients underwent TURBT followed by 61 Gy in 34 daily fractions of radiotherapy with concurrent cisplatin or 5-flurouracil/mitomycin C as a radiosensitiser. This study considered freedom from radical cystectomy after 3 years as a primary endpoint and freedom from distant progression at 3 and 5 years, progression to muscle invasion, disease-specific and overall survival, and safety as secondary endpoints. This was a small study involving 37 patients, 34 of whom were included in the final analysis. The authors demonstrated that this approach afforded 88% freedom from cystectomy at 3 years and an overall survival of 69% (thought to be low mainly due to the overall morbidity of the patient cohort). The rates of distant metastases were 12% at 3 years and 20% at 5 years (95% CI 4–26 at 3 years and 18–37 at 5 years) [23]. The primary endpoint of freedom from cystectomy at 3 years performed better than historic controls [23].

Survival outcomes, however, do not equate to quality of life. Radical cystectomy is a major operation that removes the bladder, the prostate, and seminal vesicles in men and the uterus, fallopian tubes, and anterior vagina in women and is associated with a 1.5–2% mortality rate in the 30-day post-operative period [24]. The long-term sequalae of urinary diversion can include metabolic acidosis, malabsorption of B12, urinary retention and hydroureteronephrosis, recurrent urinary tract infection, and renal dysfunction [24]. Conversely, bladder radiotherapy can also cause long-term adverse effects secondary to chronic fibrosis and progressive endarteritis in the pelvis, which can result in the loss of sexual function, strictures, haemorrhagic cystitis, and pain and rectal issues, such as tenesmus and faecal urgency/incontinence [25,26]. It should be acknowledged that the likelihood of long-term adverse effects for both approaches is low. Overall, patients who preserved their bladder reported better quality-of-life outcomes based on validated surveys with over 70 data points (combined results from three studies). However, the cost of trimodality treatment was significantly higher, with radical cystectomy costing USD 148757 per quality-adjusted life year versus USD 289142 of trimodality therapy using EuroQOL EQ-5D 3L values [27,28]. This was conducted in the US, so the costs are specific to that healthcare system; hence, the general applicability of these data is not guaranteed. A UK-based study suggested that patient satisfaction was generally high in terms of bladder and bowel function regardless of treatment; however, patients who preserved their bladder reported far higher levels of satisfaction in terms of sexual function [29].

Thus, radiotherapy in combination with chemotherapy is an effective therapy for bladder cancer and without the morbidity associated with radical cystectomy. These treatments provide a better quality of life although they do need more invasive monitoring compared with patients who opt for radical cystectomy. Specifically, patients who preserve their bladder will need cystoscopy as part of their monitoring, but all patients will receive scans and blood tests.

## 3. Radiotherapy Delivery

Radiotherapy does not currently form part of the SOC for NMIBC, and there are currently only two prospective studies that potentially inform radiotherapy NMIBC regimes. The first, published in 2007, looked back over the preceding 11 years and compared patients treated with 60 Gy in 30 fractions to a mixed BCG and “watchful waiting” cohort. Some criticisms of this study included the use of a mixed cohort as a comparator and the fact that 18% of the radiotherapy cohort was not treated, which collectively meant that this study had not significantly influenced practise [21,30]. The NRG Oncology/RTOG 0926 NMIBC phase II trial combined radiosensitisation with 61.2 Gy in 34 fractions, yielding comparable results to cystectomy [23]. Whilst limited conclusions can be drawn from these studies, these regimes are comparable to those used in MIBC, and it would be reasonable to mirror these pending definitive studies.

For MIBC, most studies delivered a conventional fractionation regime of 64 Gy in 32 fractions over 6.5 weeks. An individual patient data meta-analysis of 782 patients from two phase III trials in MIBC, which compared 64 Gy in 32 fractions over 6.5 weeks with a moderately hypofractionated regime of 55 Gy in 20 fractions over 4 weeks, concluded that the hypofractionated regime was superior for loco-regional control (HR 0.71 (95% CI 0.52–0.96) with no significant difference in overall survival. Previously, it was thought that hypofractionated regimes are associated with an increased risk of normal tissue damage; however, no differences in late toxicity were observed [31]. Despite the limitations of drawing comparisons between studies which were not designed to be analysed together, the obvious benefits both in terms of healthcare costs and patient wellbeing means that a hypofractionated regime is now the standard of care [31].

## 4. Avenues for Radiotherapy Optimisation

A challenge for bladder radiotherapy is the change in bladder volume due to the variation in filling, causing inter- and intra-fraction differences despite pre-fraction voiding. Organs at risk of normal tissue toxicity such as the rectum also vary in volume over time. Adaptive radiotherapy planning has the potential to improve both tumour dosing and minimise side effects by accommodating to anatomical changes in real time. To achieve this regime, two workflows were successfully implemented. The first is the “plan of the day”. This method uses information from cone beam computed tomography (CT), which takes three-dimensional soft tissue images. At each fraction, an image is taken prior to treatment. This is used to select an optimal treatment plan from a library of patient-specific treatment plans that best match the pre-treatment image. This approach has the potential to reduce the treatment margins needed due to the greater accuracy afforded [32]. Whilst there may be improved coverage with this method, it does increase treatment times and complexity and requires trained personnel to implement the workflow. The other approach for adaptive radiotherapy is the “composite method”, which uses the cone beam CT scans from the first 3–5 fractions to predict the margins for future fractions rather than using population-based margins [33]. The UK RAIDER study evaluated a “plan of the day” approach. The study found that there was no significant difference in late toxicity between the standard-of-care radiotherapy arm and the plan-of-the-day arm. It is likely that the implementation of daily image-guided radiotherapy during the recruitment of the trial affected the impact of the plan of the day [34]. Evolving approaches in this area include the use of linear accelerators capable of real-time planning, where the pre-treatment image is used to produce a daily customised radiotherapy plan [35].

Brachytherapy provides the opportunity for an increased dose of radiotherapy to be delivered to the tumour itself rather than to the whole bladder. However, this is only available in selected centres and has never been evaluated within a randomised control trial [35].

## 5. Current Use of Combination Therapy

Combining radiotherapy with systemic treatments improves the response to treatment compared to radiotherapy alone. This approach exploits multiple vulnerabilities in the tumour and can thus be more effective than treatments used in isolation. The other advantage of combination therapies is the possibility of a synergistic effect between the different treatments. The disadvantages of combining treatments range from practical concerns like a greater number of hospital appointments to worsening side effects and increasing morbidity.

## 6. Radiosensitisation

Radiotherapy exerts its effects via DNA damage, and this can occur in several ways. If the radiation interacts with the DNA, it can result in an ionisation event, or it can interact with water, resulting in the generation of high-energy electrons that create hydroxyl radicals (atoms with an unpaired valance electron), which, in turn, cause DNA damage. This kind of damage is relatively easily repaired by the tumour cell. However, if these radicals interact with molecular oxygen, a peroxyl radical is generated, and peroxyl-mediated DNA damage is much more challenging for the cell to repair. This results in a 3-fold increased efficacy of radiotherapy in the presence of oxygen, known as the oxygen enhancement ratio [36]. This DNA damage can take a variety of forms, specifically base and sugar damage, as well as single-stranded and double-stranded breaks. Double-stranded breaks are most hazardous for cells as they cause genomic instability, which, if unrepaired, result in cell cycle checkpoint pauses, leading to cell death [37].

Tumours evade the effects of radiotherapy through a variety of mechanisms, and targeting these can have a radio-sensitising effect. One potential mechanism of radio-resistance involves DNA damage repair pathways, as surviving radiotherapy necessitates repair of radiotherapy-induced DNA damage. There are many detailed reviews of DNA damage response, but, in brief, to survive radiation-induced DNA damage, the cell needs to be able to repair the DNA damage, which involves the recruitment of repair factors as well as signalling to the cell to prevent further cell division during this time. If the cell is unable to make these repairs, due to failures in these pathways or due to overwhelming levels of damage, then cell death occurs [38].

Another approach is targeting hypoxia. The chaotic growth of tumours means that the blood supply to this tissue is less organised than in healthy tissues; this under-perfusion results in a hypoxic microenvironment, which reduces the efficacy of radiotherapy due to the reduced availability of molecular oxygen. Moreover, the behaviour of the cells is fundamentally altered under hypoxia. Cells can become quiescent and stop cycling, become necrotic, or more likely to invade and metastasise to other tissues [39]. One radio-sensitising approach is to improve oxygen delivery to the tumour. The Bladder Carbogen Nicotinamide Phase 3 Randomized Trial used nicotinamide and carbogen (98% O_2_; 2% CO_2_) combined with radical radiotherapy with the view that increasing the partial pressure of oxygen in the blood stream at the point of radiotherapy delivery, as well as improving perfusion, would reduce the degree of hypoxia in the tumour. This trial, which included muscle-invasive and high-grade NMIBC participants, demonstrated a 10-year overall survival of 30% (95% CI 0.23–0.39) in patients receiving a radiosensitiser, but 24% (95% CI, 0.18–0.33) in patients who received radiotherapy alone [40].

Gemcitabine is a radiosensitiser, the exact mechanism of which remains unknown, but it has been shown to require the p38MAPK pathway [41] which is involved in growth inhibition and apoptosis [42,43]. In the GemX phase II trial, in muscle invasive bladder cancer, gemcitabine was used at one-tenth of the standard therapeutic dose and combined with hypofractionated radiotherapy (52.5 Gy in 20 fractions over 4 weeks). This study, which involved 50 patients with T2–3 N0M0 MIBC, showed a complete response rate of 88% at 3 months, a disease-specific survival of 82%, and an overall survival of 75% at 3 years, and 89% of the patients treated preserved their bladder without significant morbidity. The authors attribute the improvement in outcomes to the improved local control of the tumour as it is unlikely that, at one-tenth of the therapeutic dose, there would be significant systemic effects on micrometastases [44]. A meta-analysis of two phase II and six phase I trials of gemcitabine with radiotherapy suggests that this combination is effective and well tolerated [45].

Cisplatin is frequently used as a radiosensitiser in bladder cancer. A small, randomised control trial was conducted on 99 patients with T2 to T4b bladder cancer who were to undergo definitive radiotherapy or radiotherapy before a radical cystectomy. These patients were randomly allocated to receive either cisplatin (100 mg/m^2^ for three cycles) with concurrent radiotherapy every 2 weeks or radiotherapy alone. The outcomes in terms of distant metastasis and overall survival were the same in both cohorts, but there was a significant reduction in the frequency of pelvic recurrence in the group that received cisplatin and radiotherapy (*p* = 0.036) [46].

The largest randomised control trial conducted was BC2001, which was a multicentre phase 3 trial with 360 participants suffering from MIBC. These patients received either radiotherapy alone (55 Gy in 20 fractions over 4 weeks or 64 Gy in 32 fractions over 6.5 weeks) or radiotherapy with concurrent chemotherapy (specifically fluorouracil during fractions 1 to 5 and 16 to 20 and mitomycin C during fraction 1). The chemoradiotherapy group had better 2-year locoregional disease-free survival (hazard ratio 0.68 (5% CI, 0.48 to 0.96; *p* = 0.03)) and better 5-year overall survival (hazard ratio, 0.82 (95% CI, 0.63 to 1.09; *p* = 0.16)). It was concluded that chemoradiotherapy with fluorouracil and mitomycin C improved outcomes when compared with radiotherapy alone without increasing adverse events [47].

## 7. Radiotherapy in Combination with Immunotherapy

Radiotherapy-related upregulation of PDL-1 was demonstrated in murine models of bladder cancer, and the inhibition of PDL-1 in these models resulted in a delay in bladder cancer growth, suggesting that there might be a synergistic effect of radiotherapy and immunotherapy [48]. The focus of multiple immunotherapy–radiotherapy trials is using radiotherapy to stimulate the host immune system to an anti-tumour immune response and then to enhance or prolong this response with immunotherapeutics [49]. It is thought that radiation causes immunogenic cell death, which can lead to the abscopal effect where immune-mediated cell death leads to a sustained systemic response to immune-presented targets [49]. No convincing evidence of an abscopal effect has been found in clinical trials. It is possible that understanding and optimising this effect requires better appreciation of predictive biomarkers for immunotherapy response as well as the optimisation of the fractionation of radiotherapy in these combinations [49].

A recent phase 1 clinical trial used intravenous durvalumab (a PDL-1 monoclonal antibody) in NMIBC post BCG treatment failure. The ADAPT-BLADDER trial had 3 arms: durvalumab alone, durvalumab and BCG or durvalumab and radiotherapy. As this was a small study it is inappropriate to draw firm conclusions. However, the authors concluded that the approach was both safe and feasible to administer, with encouraging levels of complete response to treatment [50]. This study found that BCG unresponsiveness is associated with high levels of PDL-1 expression. There were some criticisms of this study pointing out that the use of the maximum tolerated dose was higher than necessary, as it increased the risk of side effects without any meaningful increase in on-target effects. This was supported by the observation that 23% of patients in the durvalumab + BCG group had to withdraw due to side effects. The radiation doses used were relatively low (18 Gy in three fractions over 1 week), which may account for the low response rate in this arm. Combining immunotherapies with radiation for NMIBC is far from straightforward with timing, dose, fractionation, and immune responsiveness contributing to the complex response [51].

## 8. Ongoing Trials

In addition to studies with published data, there are a number of immunotherapy combination studies that are still in progress (Table 2). The PREVERT trial (Bladder PREserVation by RadioTherapy and Immunotherapy in BCG Unresponsive Non-muscle Invasive Bladder Cancer) considers patients that progress following intravesical BCG treatment [52]. Building on data from muscle invasive bladder cancer, which suggests that combined chemoradiotherapy produces comparable survival rates to cystectomy [18], this study used immunotherapy together with radiotherapy. The basis for this approach is the demonstrated immunosensitising effect of radiation in other cancers via either anti-PD1-based immune stimulation through radiotherapy or PD-1-based immune stimulation increasing radiosensitivity [53]. The authors proposed a combination treatment with avelumab 5 days prior to radiotherapy and concurrently during treatment with 60–66 Gy in 30–33 fractions. The primary outcome will be high-risk recurrence-free survival at one year [52]. The HOPE-04 single arm prospective phase II trial is currently recruiting patients with high-risk/extremely high-risk non-muscle invasive bladder cancer to receive a short course of the whole bladder radiotherapy (five fractions of 5 Gy over 5–10 days) as well as toripalimab (a PD-1 targeted monoclonal antibody) [54]. Following this, there will be a focal tumour-bed boost of radiotherapy (of 9–18 Gy in 3–6 fractions), and the toripalimab will be continued for one year. The primary endpoints will be disease-free survival and safety [54].

## 9. Biomarkers

Biomarkers broadly take three forms: prognostic biomarkers, which are associated with outcomes independent of treatment; pharmacodynamic biomarkers, which are used as a measure of treatment response; and predictive biomarkers, which are defined as those most likely to benefit from a specific treatment. Current European Association of Urology guidelines stratify risk based on TNM tumour grade, the number of tumours, and the age of the patient but do not take account the genomic and proteomic characteristics of the tumour [3]. On the basis of these guidelines, a considerable proportion of bladder cancer sufferers will undergo radical cystectomy, which results in life-changing morbidity. If we are to employ radiotherapy as an alternative treatment in high-risk NMIBC, it would be helpful to be able to risk stratify the patients who would benefit from this as an alternative to cystectomy. Biomarker research has the potential to guide the decision between surgical and medical management, and prognostic biomarkers of disease severity could identify patients who would benefit from a surgical approach. In the case of rapidly progressive and aggressive disease, the risk–benefit profile would shift in favour of cystectomy. Similarly, predictive biomarkers of radiotherapy response or treatment sensitivity would identify patients who are most likely to benefit from bladder-sparing approaches. MRE11 expression has been demonstrated to be a predictive marker of survival following radiotherapy treatment of MIBC [55]. More recently, a gene signature, called radiosensitivity index, was analysed and tested on the data from the phase II CON trial in MIBC, and univariate analysis showed a non-significant association with local relapse free survival [56]. Unlike cystectomy, it is possible to overtreat with radiotherapy; in this situation, normal tissue damage is increased without further reduction in tumour burden. Real-time monitoring of treatment response could help prevent overtreatment. Ultimately, personalised risk stratification would allow for better-informed shared decision-making on the most acceptable treatment for individuals.

In addition to the need for predictive biomarkers of radiotherapy response, targeted cancer therapeutics are becoming increasingly important in the treatment of all cancers. Elucidating the molecular drivers of bladder cancer would allow for the selection of appropriate small molecule inhibitors and also predict patients who would benefit.

As with much of the research in this setting, both prognostic and predictive biomarkers are better characterised in the muscle invasive setting. However, there is evidence that NMIBC has an enhanced base excision repair capacity in comparison with the surrounding bladder tissue. The authors of this manuscript suggest that base excision repair capacity may be able to be used as a prognostic biomarker and a predictive biomarker in response to genotoxic agents [57].

NMIBC needs long-term follow-up, and the concept of surveillance without the need for invasive and relatively expensive tests such as cystoscopy is an attractive one. The ideal biomarker is easily available and amenable to repeat sampling, so biomarkers that can be evaluated using urine or blood are ideal. Recent developments in circulating DNA analysis have the potential to provide real-time monitoring of tumour burden as well as providing the opportunity to evaluate the effect of tumour genomics on response to treatment [58]. In the context of a localised disease such as NMIBC, it is likely that urinary markers would be more helpful than those isolated from blood. There are many urinary biomarkers tests for NMIBC commercially available, but these tests are seldom used clinically (for a summary of these please see Table 3 [59,60,61,62,63,64,65,66]). The main reason for this is the lack of large-scale clinical trials demonstrating that they add value and the multiple confounding factors that can alter the accuracy of the test [59]. Cytology, the analysis of tumour cells shed into the urine, can be used clinically but has a far greater sensitivity in high-grade tumours and, even in these circumstances, is recommended as an adjunct to and not a replacement for cystoscopy [5]. As a result of these limitations, none of the urinary tests developed have been able to replace cystoscopy for NMIBC surveillance.

In summary, biomarkers could enhance every aspect of NMIBC treatment. Robust prognostic biomarkers could select patients for aggressive treatment and identify those who are more likely to require surgical approaches. Predictive biomarkers could identify patients who are most likely to benefit from specific treatment approaches, and pharmacokinetic biomarkers could monitor treatment response. The stage at which the biomarker is employed would dictate sampling modality, and tissue biomarkers could be used for the selection of the initial treatment strategy, but any pharmacokinetic biomarkers or those involved in disease surveillance would need to be able to sample from blood or urine.

## 10. Conclusions

In muscle invasive bladder cancer, radiotherapy is used to good effect with comparable outcomes to radical cystectomy while avoiding the associated morbidity. Bladder-sparing therapy allows for superior patient experience and the opportunity for personalised shared decision-making. Whilst radiotherapy is not the standard of care in NMIBC, much can be learnt from the experience in muscle invasive bladder cancer.

Improvements in technology resulted in more precise radiotherapy. The combination of radiotherapy with radiosensitisers may further improve efficacy in NMIBC. Biomarker development will enable robust patient stratification and more informed treatment choices. The challenge for the future of NMIBC treatment is how best to employ these developments to capitalise on the promise of radiotherapy.

## Figures and Tables

**Table 1:** European Association of Urology risk stratification guidelines for NMIBC [3].

Risk	Low Risk	Intermediate Risk	High Risk	Very High Risk
Features	Primary, single TaT1LG/G1 with no risk factors Primary TaLG/G1 tumour without CIS with no more than 1 risk factor	Patients without CIS not in other groups	All CIS unless very high risk All T1G3 unless very high risk TaG2/T1G1 no CIS and 3 risk factors TaG3 or T1LG with 2+ risk factors T1G2 no CIS with 1+ risk factors	T1G2 and CIS with 2+ risk factors T1G3 and CIS with 1+ risk factor TaG3 and CIS with 3 risk factors T1G3 no CIS with 3 risk factors
5-year progression risk	0.93% (95% CI 0.49–1.7%)	4.9% (95% CI: 3.4–7.0%)	9.6% (95% CI: 7.4–12%)	40% (95% CI 29–54%)
Treatment	TURBT and single installation of BCG	TURBT and induction intravesical chemotherapy with or without maintenance for one year	Radical cystectomy or TURBT and intravesical BCG for 1 to 3 years	Radical cystectomy or TURBT and intravesical BCG for 1 to 3 years if radical cystectomy not possible
Additional risk factors: aged over 70, multiple papillary tumours, tumour diameter greater than 3 cm. LG—low grade G1—grade 1 G2—grade 2 G3—grade 3 CIS—carcinoma in situ TURBT—transurethral resection of bladder tumour CI—confidence interval				

**Table 2:** Immunotherapy and radiotherapy combination trials in NMIBC [50,52,54].

Trial	Patient Group	Trial Type	Number of Patients	Treatments	Primary Endpoint
PREVERT	Progression following intravesical BCG	Phase II single arm	67	Avelumab + EBRT in 60–66 Gy (30–33#)	Recurrence-free survival at 1 year
HOPE-04	High-risk/extremely high-risk NMIBC	Single arm prospective phase II	55	25 Gy in 5# followed by tumour bed boost (9–18 Gy in 3–6#) with concomitant toripalimab for 1 year	1 year disease-free survival Safety
ADAPT-BLADDER	NMIBC post BCG treatment failure	Phase I multi-arm	32	Durvalumab Durvalumab and BCG Durvalumab and EBRT	Recommended phase 2 dose Secondary endpoint complete response at 1 year

**Table 3:** Comparison of commercially available urinary biomarker assays in NMIBC.

Test	Analyte	Method	Approval		Diagnosis	NMIBC Surveillance	Limitations
			FDA	CE			
VisioCyt	Cells	Digital fluorescence using an algorithm			sensitivity > cytology	Not compared	No head to head comparisons
XpertBladder	mRNA	RT-PCR of ABL1, CRH, IGF2, UPK1B, ANXA10)			sensitivity > cytology	sensitivity > cytology	No dedicated prospective studies
Urodiag	DNA	RT-PCR of FGFR mutations and methylation of HS3ST2, SEPTIN9, SLIT2			Not compared	Not compared	No head to head comparison with cytology
Cxbladder	mRNA	RTPCR of MDX, HOXA13, CDC2, IGFBP5, CXCR2			sensitivity > cytology but specificity < cytology	sensitivity > cytology	No evidence with HG tumours,
ADXBLADDER	protein	ELISA for MDM5			sensitivity > cytology specificity < cytology	sensitivity > cytology	Contamination with vaginal flora or urinary stones can lead to false positives
BTAstat/BTA TRAK	protein	ELISA for CFHrP			sensitivity > cytology specificity < cytology		Test affected by haematuria
NMP22 BladderChek Test	protein	ELISA for NMP22			Sensitivity varies according to grade of tumour but specificity < cytology	sensitivity > cytology but specificity < cytology	Influenced by infection, inflammation and haematuria
ImmunoCyt/uCyt1+	cells	Fluorescence microscopy of 19A211, LDQ10 and M344			sensitivity > cytology but specificity < cytology	sensitivity > cytology but specificity < cytology	Interpretation requires training and high interobserver variability
UroVysion Bladder Cancer Kit	DNA	FISH for aneuploidy of chromosomes 3, 7 and 17 and loss of 9p21			No concordant data	No concordant data	Interpretation needs training, needs large volumes of sample

A brief comparison of commercially available urinary biomarkers on the basis of a recent meta-analysis of independent trials of the efficacy of these biomarkers as well as brief details of the testing method. Shaded boxes indicate where the product is approved for use [59,60,61,62,63,64,65,66].

## Data Availability

No new data were created or analyzed in this study. Data sharing is not applicable to this article.
